# Efficient Catalytic Reduction of Selected Toxic Dyes by Green Biosynthesized Silver Nanoparticles Using Aqueous Leaf Extract of *Cestrum nocturnum* L.

**DOI:** 10.3390/nano12213851

**Published:** 2022-10-31

**Authors:** Pradeep Kumar, Jyoti Dixit, Amit Kumar Singh, Vishnu D. Rajput, Pooja Verma, Kavindra Nath Tiwari, Sunil Kumar Mishra, Tatiana Minkina, Saglara Mandzhieva

**Affiliations:** 1 Department of Botany, MMV, Banaras Hindu University, Varanasi 221005, India; 2 Department of Pharmaceutical Engineering and Technology, Indian Institute of Technology, Banaras Hindu University, Varanasi 221005, India; 3 Academy of Biology and Biotechnology, Southern Federal University, 344096 Rostov on Don, Russia

**Keywords:** catalytic reduction, dye, DLS, HR-TEM, HR-SEM, AgNPs

## Abstract

In the present study, the catalytic reduction of selected toxic dyes (methylene blue, 4-nitrophenol, 4-nitroaniline, and congo red) using biosynthesized green silver nanoparticles (AgNPs) of *Cestrum nocturnum* L. was successfully performed. These AgNPs are efficiently synthesized when a reaction mixture containing 5 mL of aqueous extract (3%) and 100 mL of silver nitrate (1 mM) is exposed under sunlight for 5 min. The synthesis of AgNPs was confirmed based on the change in the color of the reaction mixture from pale yellow to dark brown, with maximum absorbance at 455 nm. Obtained NPs were characterized by different techniques, i.e., FTIR, XRD, HR-TEM, HR-SEM, SAED, XRD, EDX, AFM, and DLS. Green synthesized AgNPs were nearly mono-dispersed, smooth, spherical, and crystalline in nature. The average size of the maximum number of AgNPs was 77.28 ± 2.801 nm. The reduction of dyes using a good reducing agent (NaBH_4_) was tested. A fast catalytic reduction of dyes took place within a short period of time when AgNPs were added in the reaction mixture in the presence of NaBH_4_. As a final recommendation, *Cestrum nocturnum* aqueous leaf extract-mediated AgNPs could be effectively implemented for environmental rehabilitation because of their exceptional performance. This can be utilized in the treatment of industrial wastewater through the breakdown of hazardous dyes.

## 1. Introduction

Nanotechnology has emerged as an important area of research [1]. The synthesis of nanoparticles (NPs) was motivated by their wide application in fields such as medicine, optics, sensors, and engineering [2]. Physiochemical methods are used in the synthesis of desirable NPs. These methods have limitations in the synthesis of the NPs due to their high cost, the impact of high energy, and the toxicity of the environment. In the present scenario, different metals such as Ag, Au, Pd, Cu, and Zn are used in the synthesis of nanoparticles [3]. Biosynthesized green NPs are preferred due to their low toxicity, biocompatibility, cost-effectiveness, and sustainability for the environment [4,5,6,7]. In the biosynthesis of the green NPs, algae [8], fungi [9], bacteria [10], and plant extracts [11] are utilized.

In the green synthesis of AgNPs, plant extracts derived from different natural resources are widely used [12]. These extracts contain various phytochemicals with various functional groups that act as reducing and stabilizing agents [13]. Due to these properties of plant extracts during the green synthesis of AgNPs, no toxic substances are generated, and it can be considered safe to the environment. Water is an important component of life and using in daily life from a variety of sources, including ponds, lakes, rivers, and wells. These aquatic bodies are contaminated with toxic substances such as heavy metals, dyes, pigments, pesticides, etc., through sewage and industrial effluents. Dyes and pigments are used as coloring agents in the leather, textiles, cosmetics, food, and paper industries [14]. These dyes are exploited on a large scale by the telecommunication industries for fiberoptic technology. It is also used by semiconductor industries for LED and solar cell manufacturing [15].

Dyes are toxic and non-biodegradable, causing mutagenic and carcinogenic effects [16,17]. Due to the high melting point of dyes, their decomposition might be restricted in the soil [18]. As a result of such practices, freshwater tables are contaminated, soil fertility is degraded, and the environment is contaminated. The accumulation of these hazardous compounds in the body causes reproductive toxicity, genotoxicity, mutagenicity, teratogenicity, and neurotoxicity [19]. They also induced changes in genetic material and mitochondrial dysfunction [20].

An appropriate green approach for the environmentally friendly catalytic reduction of dyes is urgently needed to reduce the load of harmful dyes in the ecosystem and to produce less hazardous degradation products of dyes. The present study is focused on the biosynthesis of green AgNPs using the aqueous leaf extract of *Cestrum nocturnum* L. and evaluating its efficacy for the catalytic reduction of synthetic dyes.

## 2. Materials and Methods

### 2.1. Chemical Reagents

Silver nitrate (AgNO_3_), methylene blue, congo red, 4-nitrophenoland 4-nitroanaline, sodium borohydride (NaBH_4_) were purchased from Merck Sigma Aldrich (Burlington, MA, USA).

### 2.2. Plant Material and Preparation of Extract

Fresh leaves of *Cestrum nocturnum* L. were collected from the campus of Banaras Hindu University, Varanasi, India. The leaves were washed, shade dried at room temperature and grounded to fine powder. The powder was stored in airtight jar at 4 °C. Twenty-five gram of the powdered were added in 100 mL deionized double distilled water and placed on magnetic stirrer at 40 °C for 60 min. The solution was filtered through Whatman filter paper No. 1. Obtained extract was stored as stock solution at 4 °C for further uses.

### 2.3. Green Synthesis of AgNPs

A reaction mixture was prepared by taking 500 µL of 3% (*v*/*v*) of *C. nocturnum* aqueous extract (CNAQ) of leaves and mixing it with 5 mL of 1 mM silver nitrate solution. The reaction mixture was exposed to sunlight for a duration of 5 min. The intensity of the incident solar light was 63,000 lx. The color of the reaction mixture changed from pale yellow color to a dark brown color after sunlight exposure. Absorbance of the reaction mixture was measured at different time interval between wavelengths of 250 and 700 nm by the UV-visible spectrophotometer (Shimadzu UV-1800, Tokyo, Japan). After the completion of the reaction, the solution was centrifuged at 12,000 rpm for 10 min. The pellets of the AgNPs were washed with deionized water to remove the impurities associated with AgNPs. It was repeated 2–4 times, and the nanoparticles were dried under vacuum and stored for further use.

### 2.4. Characterization of Green Synthesized AgNPs

Preliminarily, information regarding the AgNP biosynthesis emerged from the change in color of the reaction mixture, and further, it was confirmed on the basis of the absorbance of the reaction mixture recorded by a UV-Vis spectrophotometer (UV-1800, Simadzu Corporation, Tokyo, Japan). Particle size and polydispersity index were analyzed by Particulate Systems, Nanoplus, Shimadzu (Otsuka Electronics Co., Ltd, Osaka, Japan). Powder samples of CNAQ and AgNPs were used for FTIR spectroscopy (Bruker, Bremen, Germany) and to investigate the presence of functional groups involved in the reduction and encapsulation of Ag+ ions during the biosynthesis of the AgNPs. The XRD pattern of the AgNPs was determined by an X-ray diffractometer (Rigaku Miniflex 600, RIGAKU Corporation, Tokyo, Japan), which was equipped with a Cu Kα radiation source and Ni filter in the range of 30–90° at the scanning rate of 2° min^−1^. High-resolution transmission electron microscopy (HR-TEM) was conducted through Techni G2 20Twin (FEI Company of USA (S.E.A.) PTE, LTD, Hillsboro, OR, USA) at 200 kV. To prepare the sample, a drop of fabricated AgNPs was loaded on a carbon-coated copper grid and dried at 24 ± 2 °C for 120 min. The sample was mounted onto the specimen holder. The particle size of the AgNPs was calculated by Image J software (version 1.54). Selected area electron diffraction (SAED) was integrated with HR TEM to examine the crystalline nature of the NPs. High-resolution scanning electron microscopy (HR SEM; Nova Nano SEM 450, Thermo Fisher, Waltham, MA, USA) was used to examine the morphological characteristics of the fabricated AgNPs. For imaging, 1nA was consistently provided for 7 s at 10 kV. Energy dispersive X-ray (EDX) was integrated with HR SEM to examine the elemental makeup of the AgNPs, so that their purity could be predicted. Atomic force microscopy (NT-MDT, Moscow, Russia) was used to study the topological characteristics of green biosynthesized AgNPs in close proximity. To examine surface properties, a drop of AgNP solution was mounted over a small glass slide and dried under a table lamp (200 W). AFM images were analyzed to calculate the size distribution and roughness profile of the AgNPs using the Nova software (version 3.2.5).

### 2.5. Catalytic Reduction in Organic Dyes with Green Synthesized AgNPs

The catalytic activity of green synthesized AgNPs was analyzed for the reduction of organic dyes (methylene blue, congo red, 4-nitrophenol and 4-nitroanaline). To induce the reduction process, NaBH_4_ was used as a reducing agent. The reaction mixture was mixed thoroughly, and NaBH_4_ was used as a reducing agent. The 4 mL of stock 1 mg/mL of each dye was mixed with 200 µL of 0.5 M NaBH_4_ followed by 200 µL of AgNPs. The characteristic spectra of the respective dye were monitored at different time intervals. A control reaction was set up without AgNPs treatment for each dye, and their absorption spectra were recorded with a UV-Vis spectrophotometer (Shimadzu UV 1800, Tokyo, Japan).

## 3. Results and Discussion

### 3.1. Biosynthesis of AgNPs and Its Visualization

**Figure 1:**
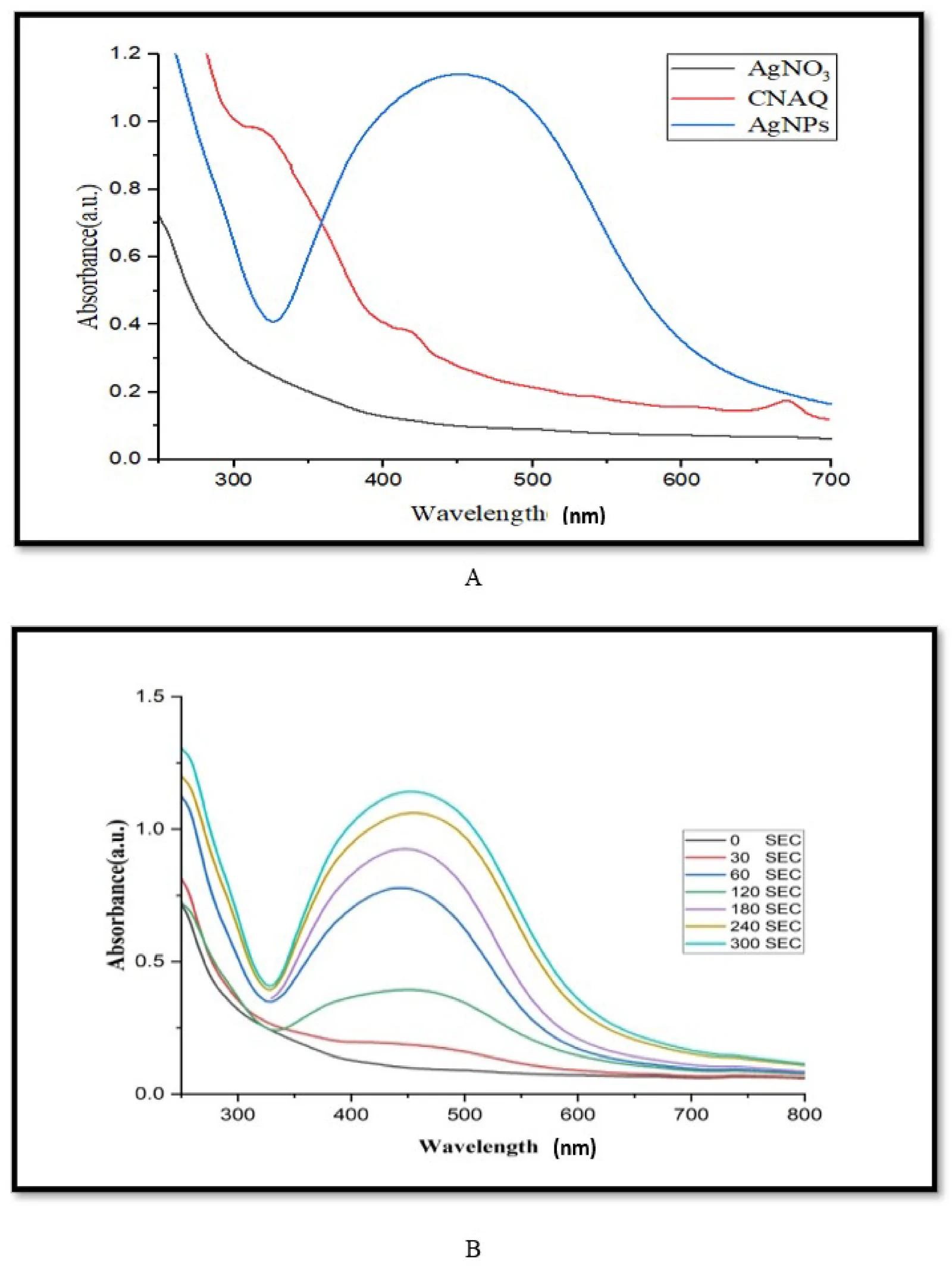
UV-Visible spectra analysis of green synthesized AgNPs. (**A**) Spectra of aqueous leaf extract of *Cestrum nocturnum* L. (CNAQ), silver nitrate and green synthesized AgNPs, (**B**) spectra of green synthesized AgNPs at different time intervals (0–300 s), reaction mixture containing 100 mL of silver nitrate (1 mM) and 5 mL inoculum (3% aqueous leaf extract).

The absorbance spectra of leaf extract of *C. nocturnum* and green synthesized AgNPs were taken under different wavelengths (Figure 1) [21]. The absorption maxima of leaf extract showed three absorbance peaks at 270, 330, and 423 nm. These peaks might be an indication of polyphenols presence, as they play an important role in the green synthesis of AgNPs [22]. Furthermore, a flask containing the reaction mixture (5 mL of extract (3%) + 100 mL of 1 mM AgNO_3_) was kept in sunlight for different time intervals. The change in color of the reaction mixture from pale yellow to brown after sunlight exposure is due to the reduction of Ag^+^ ions to metallic Ag. It confirmed the biosynthesis of AgNPs. The maximum absorbance of the reaction mixture was recorded at 250–700 nm. The absorbance between 400–500 nm suggests the biosynthesis of AgNPs.

To optimize the duration of light exposure on the biosynthesis of the AgNPs, the absorbance of the reaction mixture was recorded at 1 min intervals up to 5 min. Results revealed that a strong surface plasmon response (SPR) was recorded at 455 nm (Figure 1A). The excitation of SPR of AgNPs leads to the emergence of brown color and a rise in the intensity of absorption maxima [23]. The height of the absorbance peaks gradually increased with increasing the duration of the light exposure (Figure 1B). It suggests the yield of biosynthesized AgNPs.

### 3.2. Characterization of AgNPs

#### 3.2.1. Particle Size Distribution

**Figure 2:**
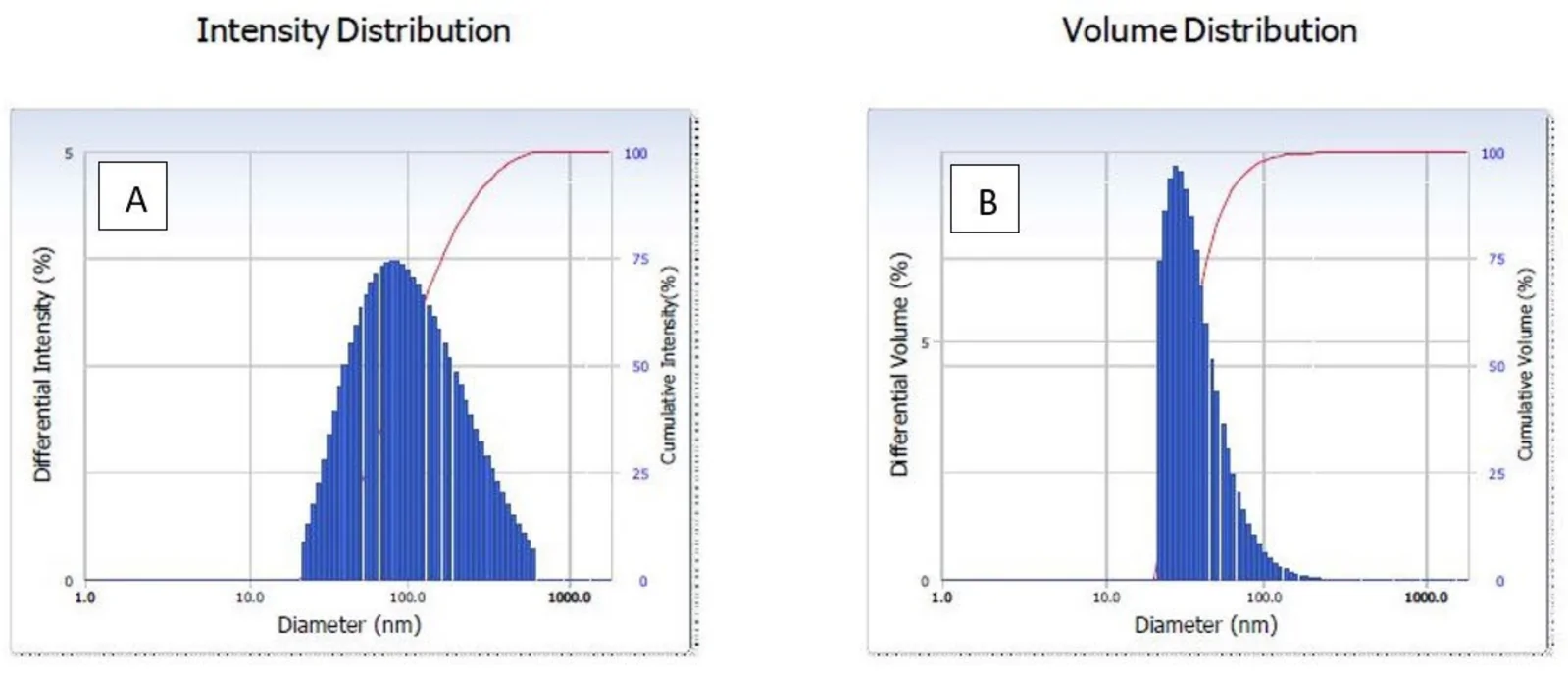
Dynamic light scattering (DLS) of green AgNPs. (**A**) Intensity, (**B**) volume distribution of green synthesized AgNPs.

The dynamic light scattering (DLS) method is applied in order to calculate the size distribution as well as the particle diameter profile of microscopic particles that are suspended in a liquid. When a laser beam strikes the circulating NPs, the intensity of the light that is initially entering the system is shifted. This modification was associated with particles. The average particle size was 91.9 nm in diameter, as shown in Figure 2A. The dynamic light scattering (DLS) particle size distribution analysis revealed the polydispersity index (pdi) of green synthesized AgNPs, which was 0.276. It indicates a nearly monodispersed nature of green synthesized AgNPs. The average volume distribution was 56 nm diameter of green synthesized AgNPs (Figure 2B).

#### 3.2.2. FTIR Spectral Analysis

**Figure 3:**
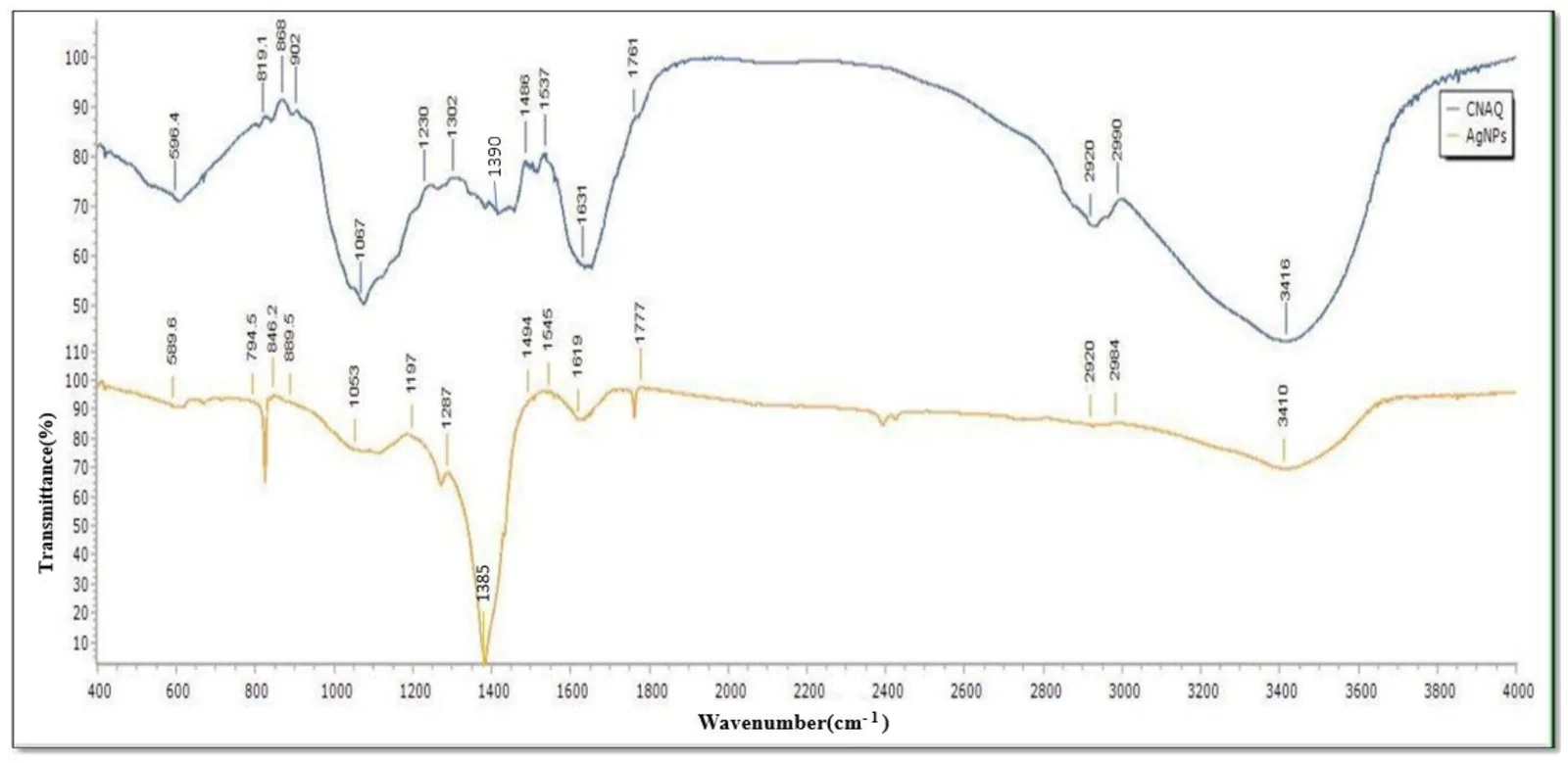
FTIR spectra of the CNAQ and AgNPs.

FTIR analysis of powder of CNAQ revealed the presence of functional groups that participate in the green synthesis of AgNPs. The FTIR spectra of CNAQ and AgNPs were recorded in range 4000–400 cm^−1^, which is attributed to the signal of stretching vibration of several functional groups (Table 1). FTIR of CNAQ represent peaks at 596, 819.1, 868, 902, 1067, 1230, 1302, 1390, 1486, 1537, 1631, 1761, 2920, 2990, and 3416 cm^−1^. In the AgNPs FTIR spectra, these peaks were shifted to 589.6, 794.5, 846.2, 889.5, 1053, 1197, 1287, 1385, 1494, 1545, 1619, 1777, 2920, 2984 and 3410 cm^−1^ (Figure 3). The broad peak at 1385 cm^−1^ detected in AgNPs corresponds to the intense vibration of NO in the NO_3_^−^. The intensity of these peaks was decreased and shifted in the spectra of the AgNPs. Similar results were reported in *Premna integrifolia* mediated AgNPs [24]. The above finding reveals that most of the peaks such as 868, 1067, 1486, 1631, and 3416 cm^−1^ represent the flavonoid group of compounds [25,26]. The reduction and shifting of these peaks suggests the involvement of these groups in the biosynthesis of the AgNPs (Table 1). It means flavonoid-rich extract of leaves participated in the biosynthesis and stabilization of the green AgNPs.

#### 3.2.3. XRD

**Figure 4:**
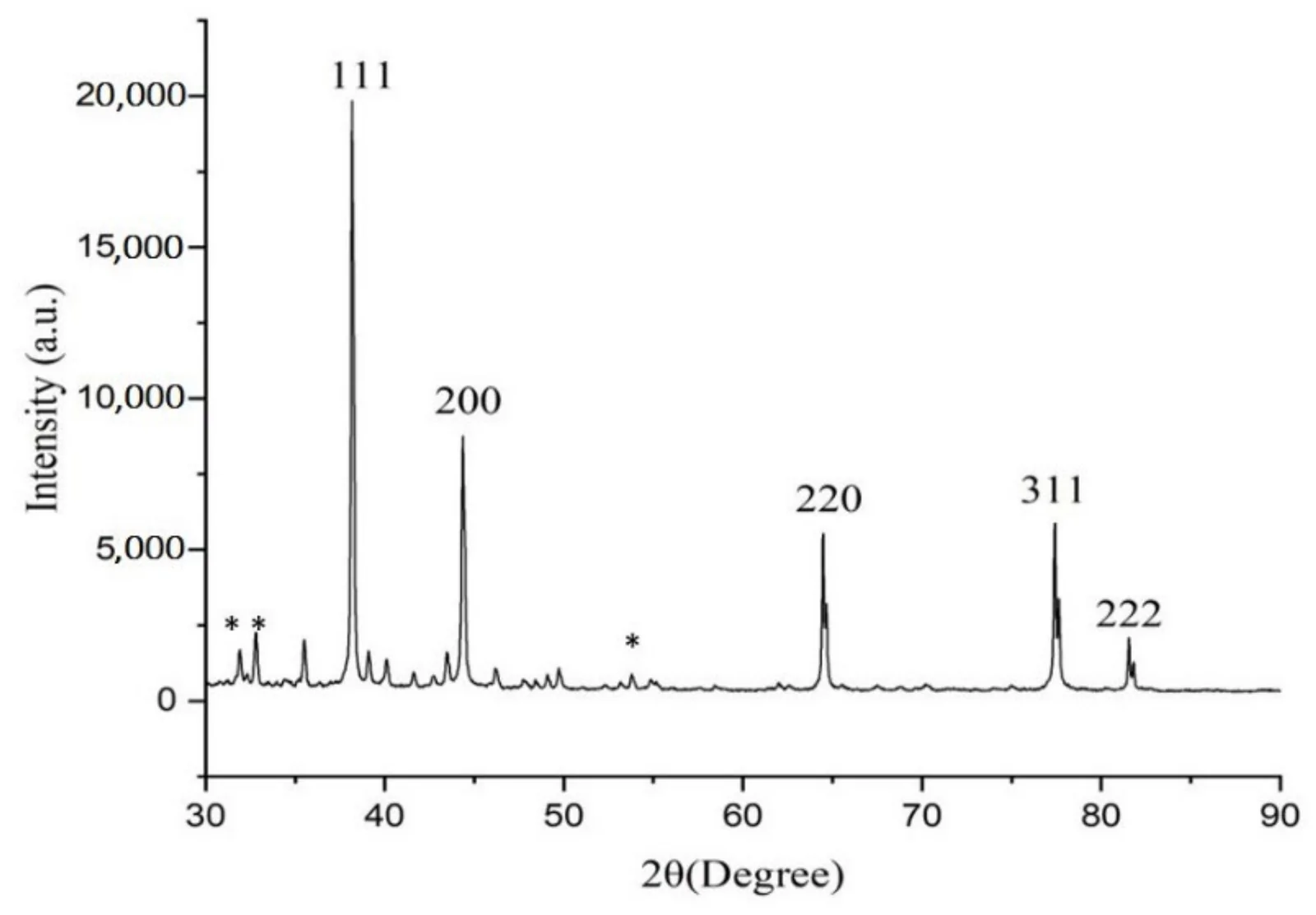
XRD pattern of AgNPs. Star (*) symbol represents small not indexed peaks such as 32.31°, 32.80° and 54.97°.

The XRD pattern of green synthesized AgNPs showed the diffraction peaks at 38.19°, 44.38°, 64.47°, 77.41°, and 81.55°, which are attributed to (111), (200), (220), (311), and (222) Bragg reflections, respectively (JCPDS file no. 00-004-0783). These Bragg reflections are corresponded to the crystalline planes of the face-centered cubic (fcc) crystal lattice of metallic silver (Figure 4). Similar crystalline peaks at 32.28°, 46.28°, 54.83°, 67.47° were observed by other workers [27,28]. The stronger planes indicate silver as a major constituent in biosynthesized green AgNPs. In the XRD spectra of AgNPs, several small not indexed peaks such as 32.31°, 32.80° and 54.97° are denoted by an (*) symbol. Peaks 32.31° and 32.80° represented organic compounds of CNAQ, while 54.97° denoted silver oxide. Similar results were reported in *Clinacanthus nutans* [29], *Ananas comosus* [30] and *Carob* mediated AgNPs [31].

#### 3.2.4. HR-TEM and SAED

**Figure 5:**
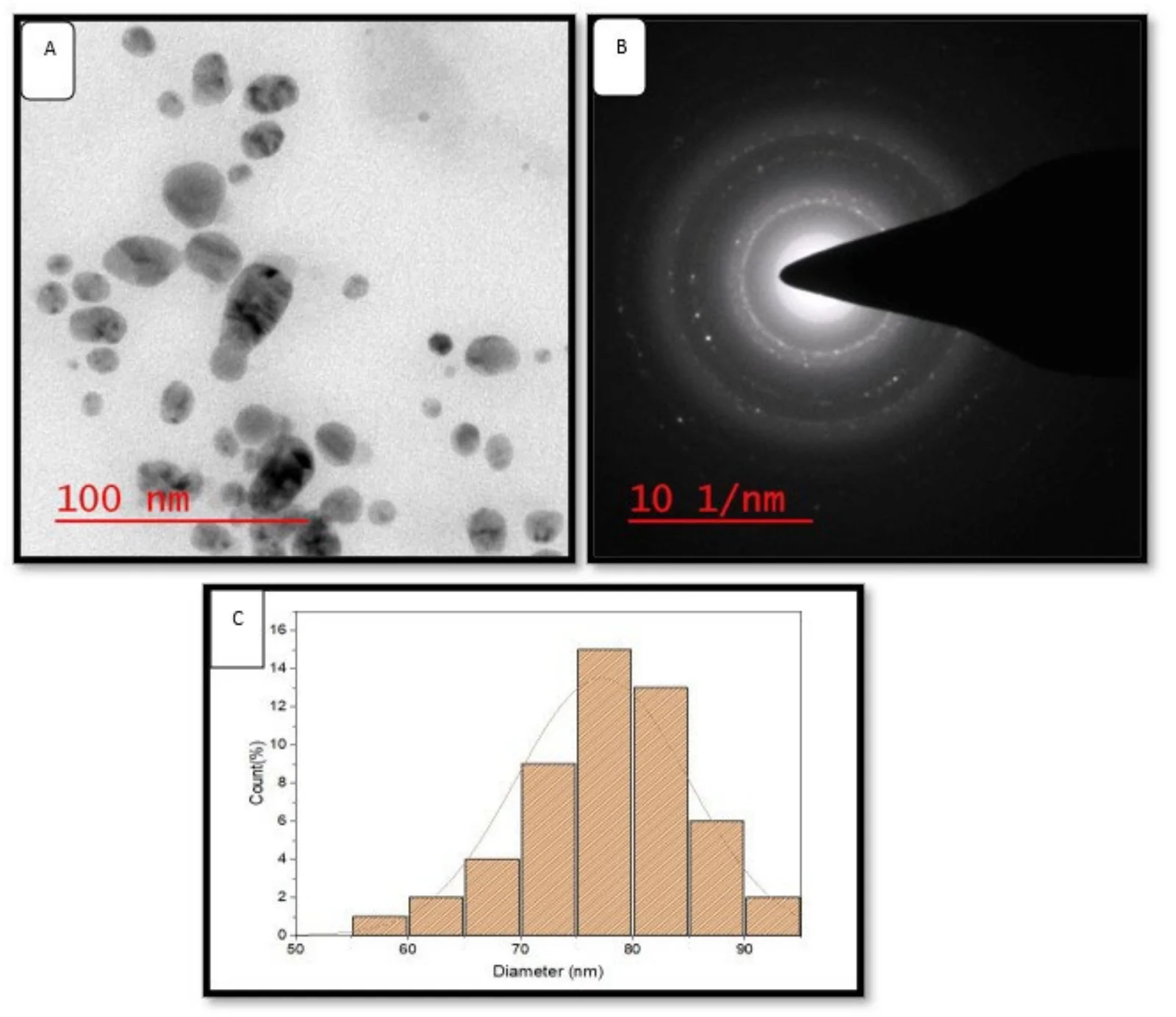
HR-TEM images of green AgNPs. (**A**) HR-TEM images of green AgNPs at 200 nm, (**B**) SEAD patterns of green AgNPs, (**C**) size distribution histogram of green AgNPs.

High-resolution transmission electron microscopy is widely used to study the size, shape and morphology of green synthesized AgNPs [15]. HR-TEM images revealed that green synthesized AgNPs were uniformly dispersed and spherical in shape (Figure 5A). Selected area electron diffraction analysis (SAED) of AgNPs depicted a bright circular ring, which revealed its highly crystalline nature (Figure 5B). SAED is commonly used to confirm the crystallinity of the AgNPs [32]. The size of synthesized AgNPs was distributed in the range of 50–95 nm (Figure 5C). The size distribution histogram (Figure 5C) confirmed the average size of the maximum number of green synthesized AgNPs was 77.28 ± 2.801 nm.

#### 3.2.5. HR-SEM and EDX

**Figure 6:**
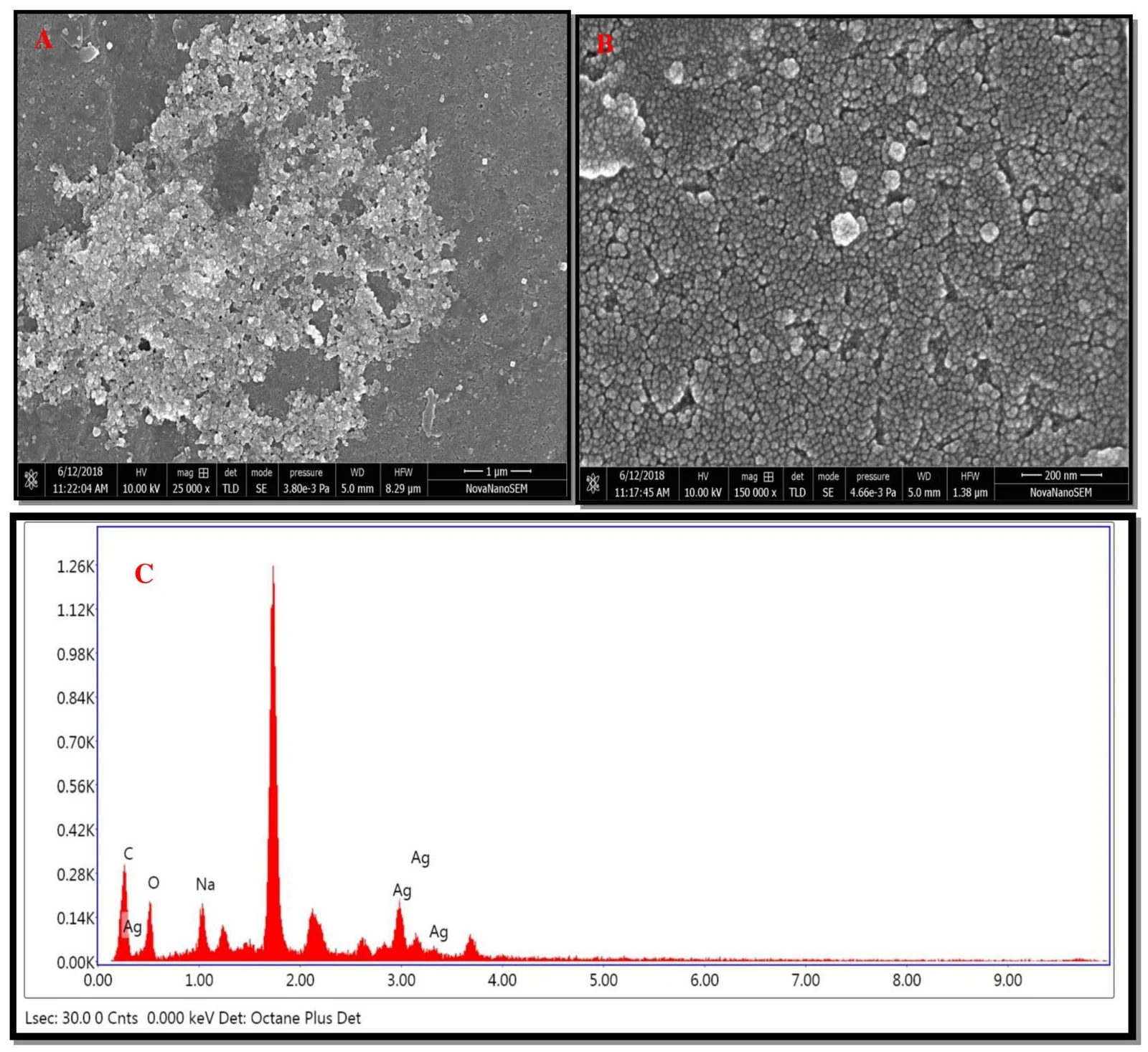
HR-SEM images of green AgNPs. (**A**) HR-SEM images of green AgNPs at 1 μm, (**B**) HR-SEM images of green AgNPs at 200 nm, (**C**) and EDX spectrum of green synthesized AgNPs.

A high-resolution scanning electron micrograph depicted the surface morphology of the green-synthesized AgNPs [33]. HR SEM (Nova Nano SEM 450, FEI Company (S.E.A.) PTE, LTD, USA) data analysis revealed that the green-synthesized AgNPs varied in size. The biosynthesized AgNPs were smooth and spherical in shape (Figure 6A). Images showed that the green-synthesized AgNPs formed aggregates (Figure 6B). The aggregation of nanoparticles might be due to proteins, carbohydrates, and glycoproteins present in the plant constituents. The EDX of the green-synthesized AgNPs showed the presence of silver (Ag). The sharp peak at 3 keV indicated the presence of metallic Ag (Figure 6C). Additional peaks (O, Na, and Si) might be because of the glass on which the sample was coated, and C due to the extract.

#### 3.2.6. AFM

**Figure 7:**
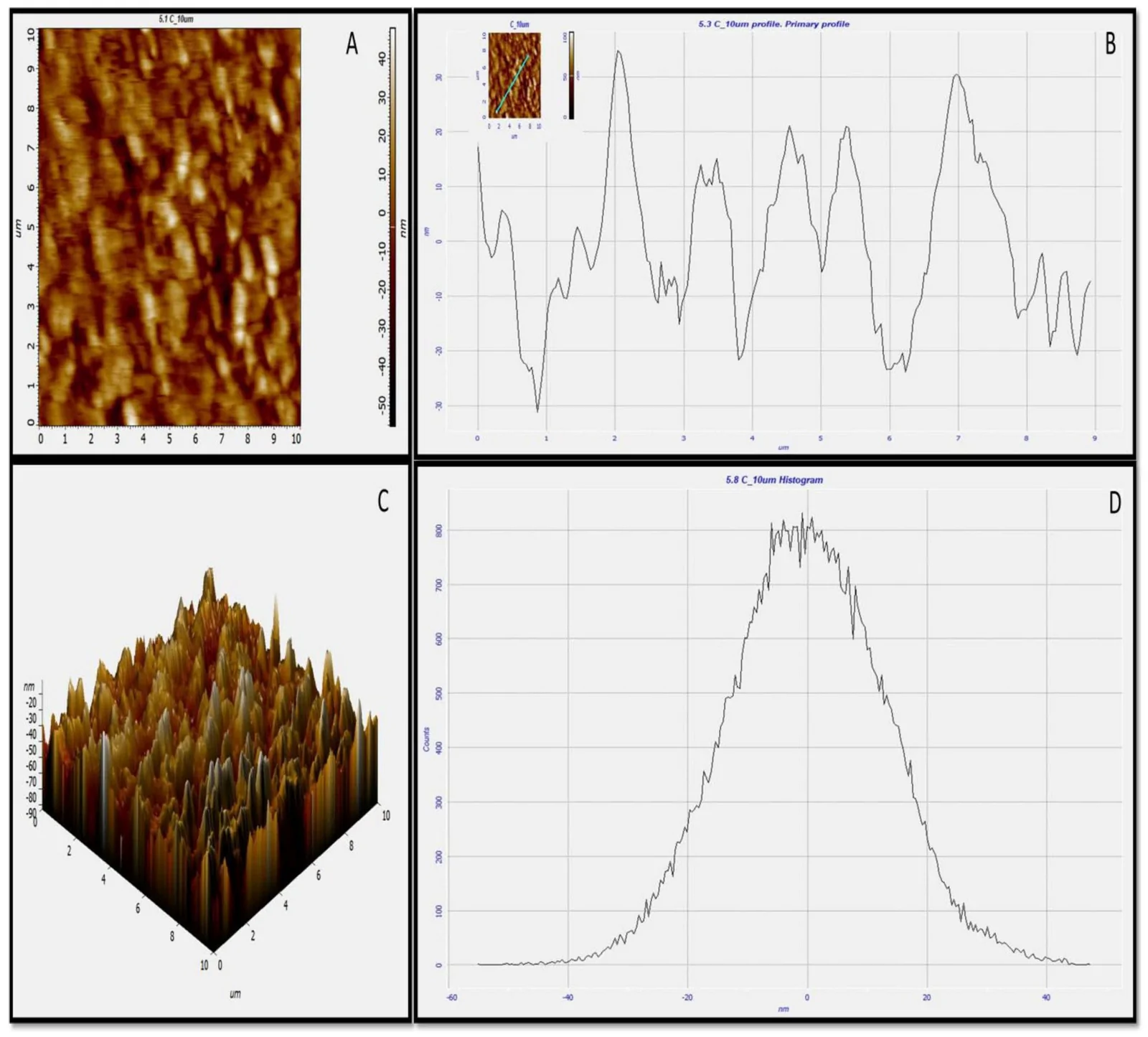
AFM images of green synthesized AgNPs. (**A**) Two-dimensional (2D) image of green AgNPs, (**B**) 2D roughness profile of green AgNPs, (**C**) 3D image of green AgNPs, (**D**) 3D roughness profile of green AgNPs.

Topographical features of green synthesized AgNPs were examined by atomic force microscopy [34]. The 2D (Figure 7A) and 3D (Figure 7C) images revealed the spherical nature of green synthesized AgNPs, which is correlated with HR-TEM and HR-SEM outcomes. The roughness of biosynthesized AgNPs was very clear, as given in Figure 7B,C. Image analysis using Nova software, including the average roughness, maximum profile peak height, and valley depth of AgNPs, was calculated. The average roughness of green synthesized AgNPs was 10.12 nm (Figure 7B). The maximum profile peak height and valley depths were 18.327 nm and 23.405 nm, respectively, in the 2D image. In the 3D image, the average roughness of green synthesized AgNPs was 14.41 nm, while the maximum profile peak height and valley depth was reported 47.73 nm and 55.07 nm, respectively (Figure 7D).

### 3.3. Mechanism of Biosynthesis of the AgNPs

**Figure 8:**
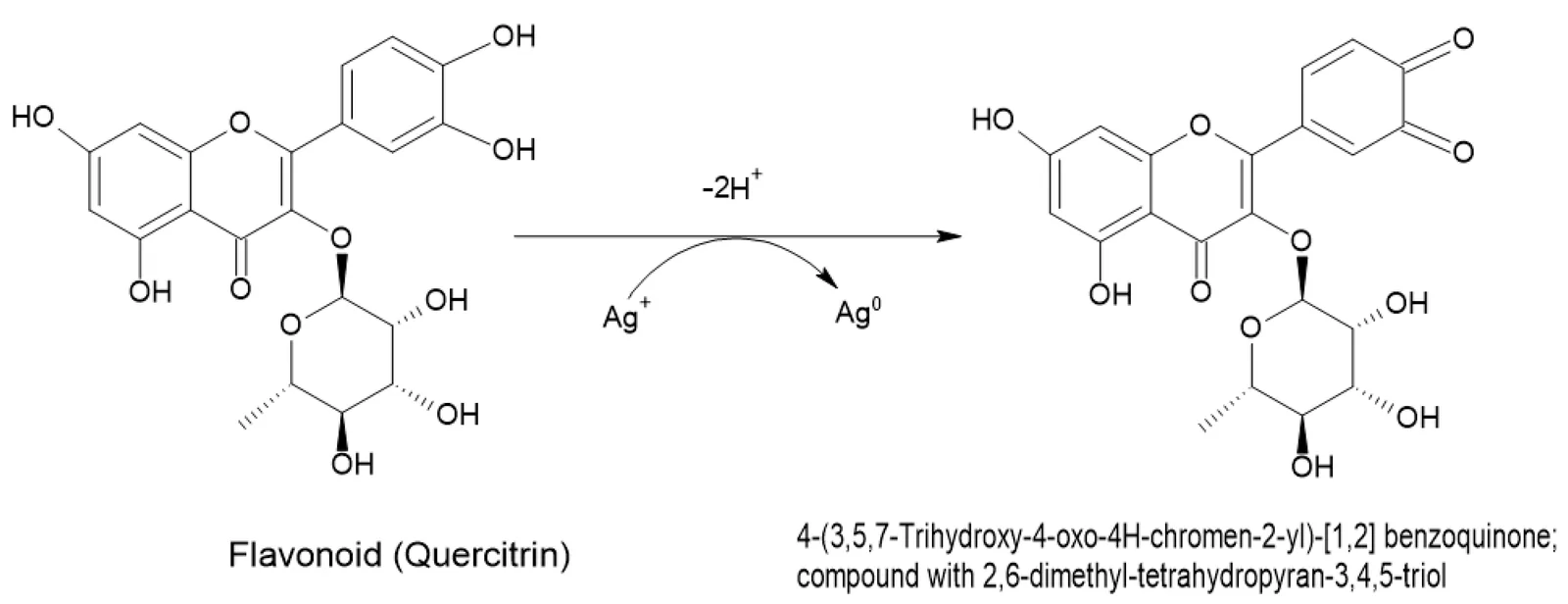
The biosynthesis mechanism of the AgNPs through involvement of the flavonoid (Quercitrin) present in CNAQ.

CNAQ is rich in many bioactive compounds such as flavonoids, steroids, glycosides, triterpenes, caffeoyl derivatives, and other aromatic compounds [35,36]. Proteins, glucose, and other bioactive components found in the leaf extract serve as strong capping agents for AgNPs formation, while flavonoids act as effective reducing agents [37,38]. A possible mechanism of the biosynthesis of AgNPs by using bioactive compounds of the CNAQ is given in Figure 8. During AgNPs biosynthesis, a reduction of Ag^+^ to Ag^0^ took place by several phytochemicals present in CNAQ, particularly flavonoids present in the extract acting as a strong reducing agent [29]. Quercitrin is an important photoactive flavonoid with a huge number of -OH groups present in the extract. The phenylpropanoid pathway produces Quercitrin, which is finally formed from phenylalanine. When kept under solar radiation, during the reaction mixture, the release of two electrons from -OH groups of Quercitrin takes place, which are used in the reduction process of Ag^+^ to Ag^0^. Furthermore, it catalyzes the biosynthesis of AgNPs. This assumption was supported by the IR examination, which revealed that the -OH groups are involved in the synthesis and stabilization of AgNPs.

The charge-transfer-induced conformational modifications in the flavonoids (Quercitrin) during the reduction of Ag^+^ to Ag^0^. The elimination of two hydrogen atoms of the enol form and the deduction of two electrons are capable of reducing 2Ag^+^ to 2Ag^0^, accelerating the synthesis of AgNPs [33].

### 3.4. Catalytic Reduction of Dyes by Green AgNPs

Dyes are colored compounds extensively used to color the products of a wide range of industries. Industrial effluents contain these dyes. Every year, approximately 7 × 10^5^ tonnes of synthetic dyes are used worldwide [39]. Dyes-based effluents cause a serious hazard to the water stream and environment due to their synthetic origin and complex molecular structure, which decreases their ability to biodegrade. The release of the effluents into the aquatic environment without sufficient treatment generates detrimental effects on the qualities of the aquatic bodies such as biological oxygen demand (BOD), chemical oxygen demand (COD), color, heavy metal content, penetration of light and other properties. Several traditional treatment techniques, such as UV-light degradation, flocculation, activated carbon sorption, redox treatment, and electrocoagulation, have been used for degradation of the effluent [40]. These techniques are costly, time consuming, and produce by-products. In order to reduce these challenges, researchers are focusing on natural substitutes for the degradation of synthetic dyes [41]. Biological treatments are the ideal techniques for improving the quality of the effluents because they are cost-effective and efficient. Using green nanoparticles might be a good way to get rid of hazardous dyes for environmental remediation.

**Figure 9:**
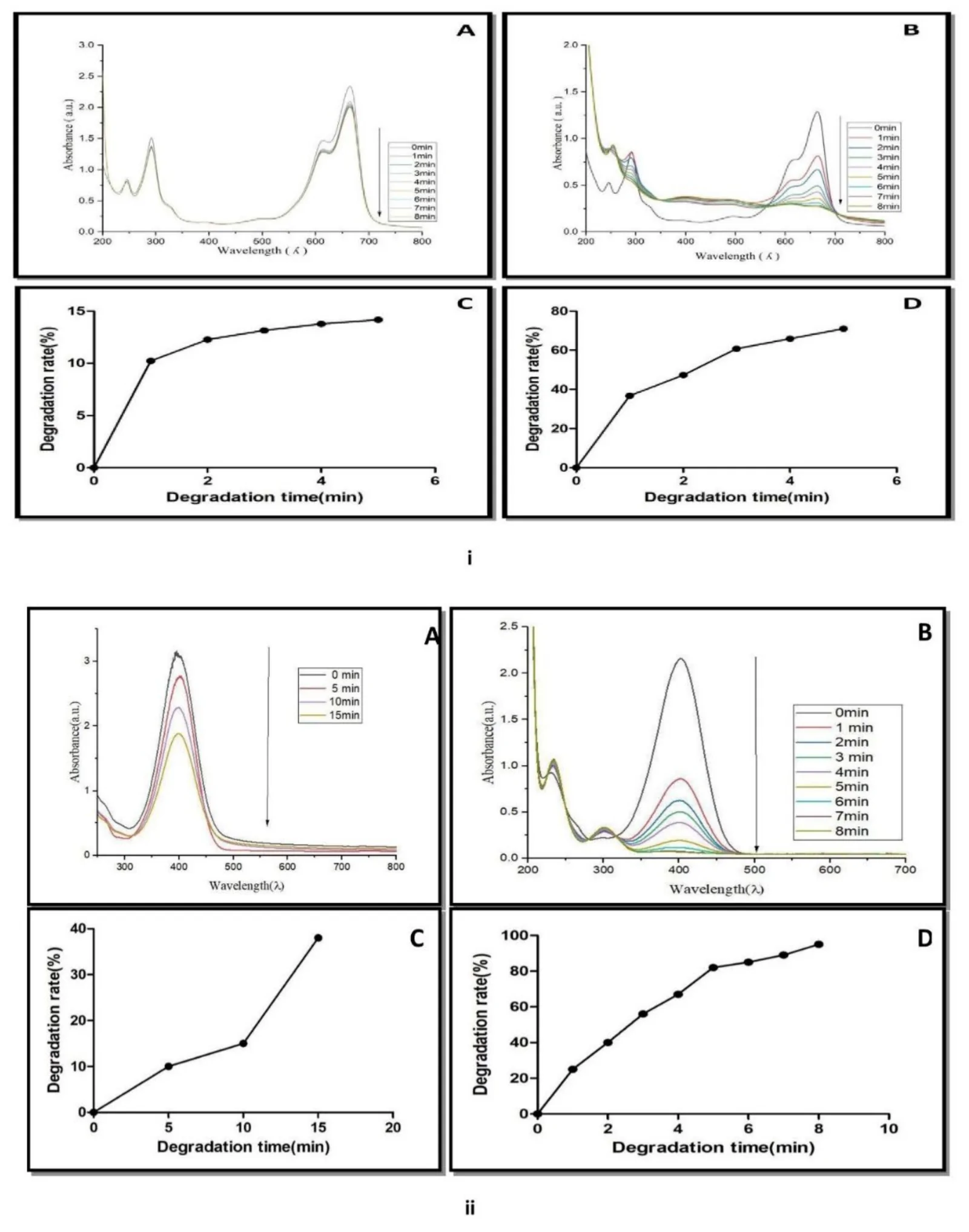
(**i**) UV-Vis spectra of methylene blue reduction in presence of (**A**) NaBH_4_, (**B**) NaBH_4_ + green biosynthesized AgNPs. (**C**) Catalytic reduction (%) of methylene blue in the presence of NaBH_4_. (**D**) Catalytic reduction (%) with NaBH_4_ + AgNPs. (**ii**) UV-Vis spectra of 4-nitro phenol reduction in the presence of (**A**) NaBH_4_, (**B**) NaBH_4_ + green biosynthesized AgNPs, (**C**) Catalytic reduction (%) with NaBH_4_. (**D**) Catalytic reduction (%) with NaBH_4_ + AgNPs.

Methylene blue belongs to the phenothiazine dye class. It decomposes at 100–110 °C [20]. It showed a maximum absorption peak at 664 nm. The control experiment containing NaBH_4_ and methylene blue solution did not show a significant change in the blue color of the solution as well as a decrease in absorption spectra with respect to time (Figure 9(iA)). The addition of AgNPs to the reaction mixture (methylene blue and NaBH_4_) completely decolorized the dye from blue color to colorless. The absorption spectra of methylene blue (664 nm) also disappeared within 8 min (Figure 9(iB)). The catalytic reduction percentage of methylene blue was 15% in the presence of NaBH_4_ (Figure 9(iC)), while the catalytic reduction percentage, was 79% within 8 min, when AgNPs was added to methylene blue and NaBH_4_ solution (Figure 9(iD)). The results showed that AgNPs in the presence of NaBH4 catalyzed the complete reduction of methylene blue into leuco methylene blue in the presence of NaBH4 [42]. Similar results were reported in AgNPs of *Thymbra* [22], *Trigonella foenum-graecum* [43], and the fruits of *Terminalia chebula* [44], indicating their nanocatalyst potential for the reduction of methylene blue.

The nanocatalytic reduction of dye at 1 mg mL^−1^ concentration was studied by observing the change in the characteristic absorption spectra of 4-nitrophenol dye. 4-Nitrophenol (C_6_H_5_NO_3_) is also known as p-nitrophenol or 4-hydroxy nitro-benzene. It is a phenolic molecule with a nitro group and a hydroxyl group at the benzene ring. 4-Nitrophenol is widely used for the production of several drugs, including acetaminophen, fungicides, and insecticides (methyl and ethyl parathion). It is also used for leather darkening and as a coloring agent [45]. When 4-nitrophenol is mixed with sodium borohydride (NaBH_4_), the absorption spectra slowly decreased within 0–15 min (Figure 9(iiA)). NaBH_4_ did not show a significant change in yellow color of 4-nitrophenol. The reduction of 4-nitrophenol by NaBH_4_ was approximately 39% (Figure 9(iiC)).

It means that 4-nitrophenol is not completely converted into 4-aminophenol. AgNPs catalyzed the fast reduction of 4-nitrophenol into 4-aminophenol when it was added to a reaction mixture containing 4-nitrophenol and NaBH_4_. After the addition of AgNPs to the reaction mixture, a rapid decline in absorption spectra was recorded (Figure 9(iiB)). A new spectrum appeared at 300 nm during the conversion. The decline of the absorption spectrum at 400 nm and the appearance of new spectra at 300 nm revealed the complete reduction of the 4-nitrophenol into 4-aminophenol within 8 min. Approximately 92% of the 4-nitrophenol is transformed into 4-aminophenol within 8 min (Figure 9(iiD)). A similar result was reported by several plant such as *Dillenia indica* bark extract [46], leaf extract of *Euphorbia heterophylla* [47] and other plant-mediated AgNPs in the reduction of 4-nitrophenol [48]. These reports concluded that the appearance of the new peak at 300 nm was due to the conversion of the -NO_2_ to -NH_2_. 4-Nitroaniline (C_6_H_6_N_2_O_2_) is another important dye known as p-nitroaniline or 1-amino-4-nitrobenzene. It is a yellow and amorphous organic dye.

It is used as a corrosion inhibitor and an intermediary in the manufacturing of colors, medicines, fuels, and poultry pharmaceuticals [49]. The catalytic reduction of 4-nitroaniline was analyzed with or without AgNPs in the presence of NaBH_4_. The maximum absorption spectrum of dye was 382 nm. NaBH_4_ was added to the dye solution, and there was also a reduction in the absorption spectrum and a slight change in the yellow color of the dye solution occurred.

**Figure 10:**
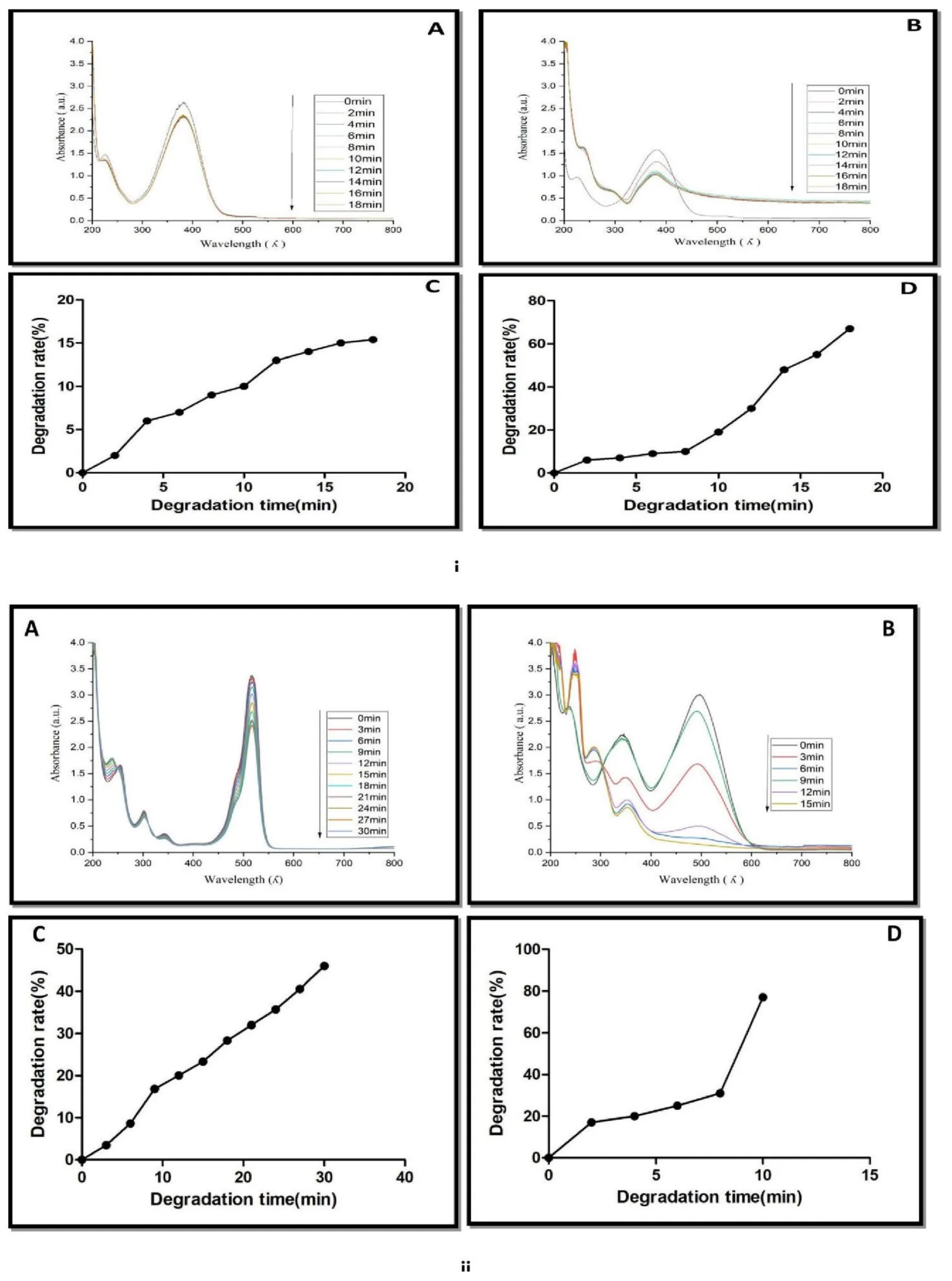
(**i**) UV-Vis spectra of 4-nitroaniline reduction in presence of (**A**) NaBH_4_, (**B**) NaBH_4_ + green biosynthesized AgNPs, (**C**) Catalytic degradation (%) with NaBH_4_. (**D**) Catalytic degradation (%) with NaBH_4_ + AgNPs. (**ii**) UV-Vis spectra of congo red reduction in presence of (**A**) NaBH_4_ and (**B**) NaBH_4_ + green biosynthesized AgNPs. (**C**) Catalytic degradation (%) with NaBH_4_. (**D**) Catalytic degradation (%) with NaBH_4_ + AgNPs.

This suggests the slow catalytic reduction of the 4-nitroaniline in the presence of NaBH_4_ (Figure 10(iA)). It was also noted that there was no significant change in the absorption spectrum of 4-nitroaniline within 30 min. The addition of AgNPs in the mixture of dye and NaBH_4_ solution resulted in the disappearance of the absorption spectra at 382 nm and the decolorization of 4-nitroaniline (Figure 10(iB)). The percentage of 4-nitroaniline reduction was 18% by NaBH_4_ (Figure 10(iC)), while its reduction was increased to 78% by NaBH_4_ and AgNPs (Figure 10(iD)). Similar results were reported in *Citrus aurantifolia* peel extract derived silver nanoparticles.

**Figure 11:**
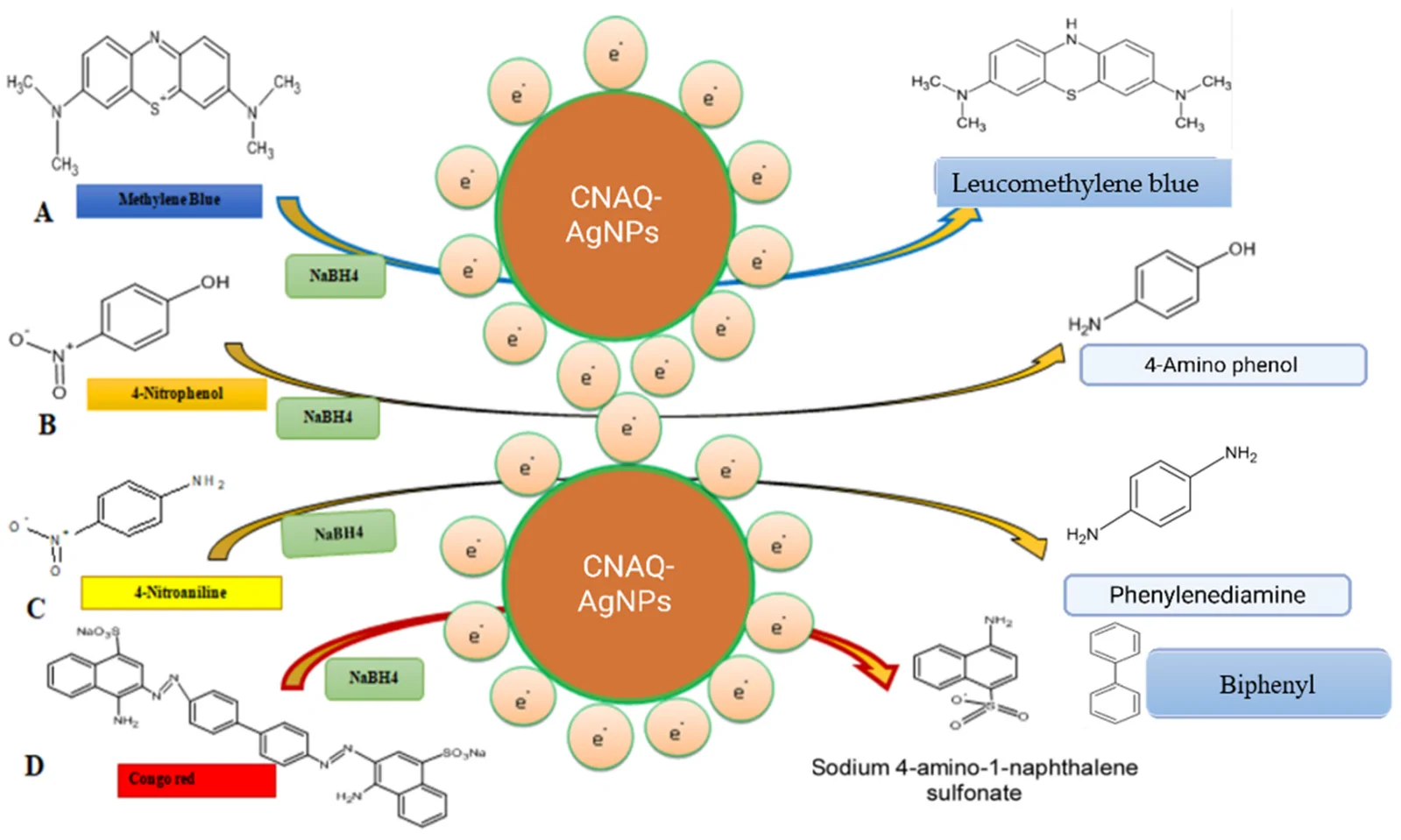
Reduction mechanism of toxic dyes and their catalytic reduction products through the involvement of the AgNPs. (**A**) methylene blue, (**B**) 4-nitrophenol, (**C**) 4-nitroanaline, (**D**) congo red.

The change in the color of the dye from yellow to colorless was observed after the addition of green biosynthesized nanoparticles. It means that the conversion of 4-nitroanaline took place into 4-aminoaniline. A similar result regarding the reduction of 4-nitroanaline by biosynthesizing green nanoparticles was reported by Sarvalkar et al. (2021) [50]. Congo red (C_32_H_22_N_6_Na_2_O_6_S_2_) belongs to the azo dye class. It showed two characteristic absorption peaks at 490 nm and 330 nm (Figure 10(iiA,B)). The formation of two different absorption peaks at 490 nm and 330 nm is due to electron transitions from π→π* and n→π* respectively. The catalytic reduction of congo red was observed for up to 30 min in the presence of NaBH_4_ (Figure 10(iiA)). Results revealed that the change in absorption spectrum was very slow. It suggests a very slow catalytic reduction of congo red by NaBH_4_ (Figure 10(iiC)). The reduction of dye in the presence of AgNPs was monitored, and a change in absorption spectrum was noted between 0 and 15 min (Figure 10(iiB)). The addition of AgNPs to the reaction mixture of congo red and NaBH_4_ synergistically enhanced the reduction of dye, leading to a change in the color of the reaction mixture from red to colorless within 15 min. It also demonstrated the disappearance of both absorption peaks (490 nm and 330 nm) and dye reduction up to 80% within 15 min (Figure 10(iiD)). Similar results were reported in *Amaranthus gangeticus* [51] and *Anacardium occidentale* [52] extract-mediated AgNPs. The reduction mechanism of methylene blue, 4-nitrophenol, 4-nitroanaline and congo red are shown in Figure 11A–D.

It might be possible that AgNPs mediated the breakage of the -N=N- double bond of the chromophore group of azo dye into sodium (4 amino-3-diazenenylnapthalene) sulfonate, phenyl, and benzene due to which the reaction mixture turned colorless. The result obtained was in accordance with the catalytic reduction of a variety of dyes in water by recyclable nano catalysts [22]. The reduction of dye was a thermodynamically spontaneous reaction that required large free energy. Due to the large transition energy gap between an electron donor (NaBH_4_) and an electron acceptor (dye), no electron transfer was facilitated [53]. Silver nanoparticles catalyzed heterogeneous catalysis reactions. As a result, green synthesized silver nanoparticles have a high surface to volume ratio. Thus, these nanoparticles have been used for a variety of uses [54]. The mechanism of catalytic reduction of dye through silver nanoparticles involves electron transfer and the least activation energy [55].

For the purpose of reducing the dyes, AgNPs serve as electron relays, allowing for the transfer of electrons from the BH_4_-ion toward the dye. At the same time that the NPs’ surfaces were becoming saturated with BH4-ions, electron transfer from the ion to the dye was taking place [56]. Silver nanoparticles assisted the transfer of electrons from reducing agent BH_4_- to dye and lead to the disintegration of the chromophore structure of dye into small molecules [57]. They reduce the kinetic barrier between the nucleophilic NaBH_4_ and electrophilic dye as a redox catalyst.

It has been observed that certain additional nanomaterials, such as Au/g-C_3_N_4_/Co_3_O_4_ [58], Au/g-C_3_N_4_/Cu_2_O [59], and CeO_2_/g-C_3_N_4_ [60], have the ability to efficiently catalyze the reduction of chromium (Cr^6+^), Bisphenol A (BPA), and oxytetracycline hydrochloride, in addition to the formation of hydrogen.

## 4. Conclusions

The nanocatalytic reduction of harmful dyes methylene blue, congo red, 4-nitrophenol, and 4-nitroaniline was achieved through biosynthesized green silver nanoparticles using aqueous leaf extract of *Cestrum nocturnum* L. Based on HR-SEM and HR-TEM analysis, most of the biosynthesized green AgNPs were mostly spherical in shape. The size distribution of AgNPs was calculated with the help of DLS and TEM, which were in the range of 50–95 nm. The catalytic reduction of the selected dyes in the presence of the good reducing agent NaBH_4_ was slow, which was monitored by taking absorption spectra. Biosynthesized green silver nanoparticles, when added to the reaction mixture containing NaBH_4_ and dye solution, decolorized the dyes within a short time. The correlation between the decolorization and disintegration of chromophore complex structures of the dyes was validated by the UV-Visible absorption spectra of the catalytic reduction reactions. After adding the AgNPs as a catalyst, more than 90% of the dye 4-nitrophenol and congo red were degraded within 8 and 15 min, respectively. Reduction of the dyes 4-nitroaniline and methylene blue up to 78–79% was completed in 8 and 18 min, respectively. The above results suggest that the photoinduced AgNPs have excellent catalytic activity. The catalytic reduction efficiency of biosynthesized AgNPs is supposed to be due to the presence of various primary and secondary metabolites. Thus, we can conclude that the enhanced ability of biosynthesized AgNPs for the catalytic reduction of toxic dyes will help in recycling the aquatic sources that are contaminated with toxic dyes in the future.

## Figures and Tables

**Table 1:** FTIR profiling of CNAQ and AgNPs.

Frequency (cm^−1^)	Peak Wavenumber		Functional Group /Type of Bond	Compounds
	CNAQ	AgNPs		
720–590	596.4	589.5	O-H out-of-plane bend	Hydroxy compounds
1000–600	902 868 819.1	889.5 846.2 794.5	C-H stretching C-H stretching C-H stretching	Alkenes Alkenes Alkenes
1140–1070	1067	1053	C-O stretching	Cyclic ether and oxy group of compounds
1250–1020	1230	1197	C-N- stretching	Aliphatic amines
1420–1300	1302	1287	Carboxylate	Carbonyl compounds
1490–1410	1486	1494	Carbonate ion	Inorganic compounds
1370–1390	1390	1385	N-O stretching	Nitrate ion
1625–1440	1537	1545	C=C stretching	Aromatic compounds
1680–1600	1631	1619	C=C stretching	Alkenes
2000–1650	1761	1777	C-H bending	Aromatic compounds
3200–2500	2990 2920	2984 2920	O-H stretching O-H stretching	Carboxylic acid Carboxylic acid
3550–3200	3416	3410	O-H stretching	Phenol, hydroxy compound

## Data Availability

Not applicable.
