# Correction: Kumar et al. Efficient Catalytic Reduction of Selected Toxic Dyes by Green Biosynthesized Silver Nanoparticles Using Aqueous Leaf Extract of *Cestrum nocturnum* L. *Nanomaterials* 2022, *12*, 3851

**DOI:** 10.3390/nano16171056

**Published:** 2026-08-25

**Authors:** Pradeep Kumar, Jyoti Dixit, Amit Kumar Singh, Vishnu D. Rajput, Pooja Verma, Kavindra Nath Tiwari, Sunil Kumar Mishra, Tatiana Minkina, Saglara Mandzhieva

**Affiliations:** 1 Department of Botany, MMV, Banaras Hindu University, Varanasi 221005, India; pradeep.kumar@bhu.ac.in (P.K.); dixit.jyoti12@gmail.com (J.D.); poojaverma2203@gmail.com (P.V.); 2 Department of Pharmaceutical Engineering and Technology, Indian Institute of Technology, Banaras Hindu University, Varanasi 221005, India; amitsinghbais7@gmail.com (A.K.S.); skmishra.phe@itbhu.ac.in (S.K.M.); 3 Academy of Biology and Biotechnology, Southern Federal University, 344096 Rostov on Don, Russia; tminkina@mail.ru (T.M.); msaglara@mail.ru (S.M.)

## Title Correction

In the original publication [1], there was an error in the title. The title should read:

Efficient Catalytic Reduction of Selected Toxic Dyes by Green Biosynthesized Silver Nanoparticles Using Aqueous Leaf Extract of *Cestrum nocturnum* L.

## Text Correction

In the originally published version of this manuscript, an error was identified in the main text. Specifically, the word “degradation” was incorrectly used and has now been revised to “reduction” where appropriate in the main text of the manuscript.

In the originally published version of this manuscript, an error was identified in the main text. Specifically, the spelling of the compound name “quercetrin” was incorrectly used and has now been revised to “Quercitrin” throughout the main text of the manuscript.

There was an error in the original publication. The manufacturer’s data were not correct. A correction has been made to Section 2. Materials and Methods, 2.4. Characterization of Green Synthesized AgNPs, Paragraph 1.

Preliminarily, information regarding the AgNP biosynthesis emerged from the change in color of the reaction mixture, and further, it was confirmed on the basis of the absorbance of the reaction mixture recorded by a UV-Vis spectrophotometer (UV-1800, Simadzu Corporation, Tokyo, Japan). Particle size and polydispersity index were analyzed by Particulate Systems, Nanoplus, Shimadzu (Otsuka Electronics Co., Ltd, Osaka, Japan). Powder samples of CNAQ and AgNPs were used for FTIR spectroscopy (Bruker, Bremen, Germany) and to investigate the presence of functional groups involved in the reduction and encapsulation of Ag+ ions during the biosynthesis of the AgNPs. The XRD pattern of the AgNPs was determined by an X-ray diffractometer (Rigaku Miniflex 600, RIGAKU Corporation, Tokyo, Japan), which was equipped with a Cu Kα radiation source and Ni filter in the range of 30–90° at the scanning rate of 2° min^−1^. High-resolution transmission electron microscopy (HR-TEM) was conducted through Techni G2 20Twin (FEI Company of USA (S.E.A.) PTE, LTD, Hillsboro, OR, USA) at 200 kV. To prepare the sample, a drop of fabricated AgNPs was loaded on a carbon-coated copper grid and dried at 24 ± 2 °C for 120 min. The sample was mounted onto the specimen holder. The particle size of the AgNPs was calculated by Image J software (version 1.54). Selected area electron diffraction (SAED) was integrated with HR TEM to examine the crystalline nature of the NPs. High-resolution scanning electron microscopy (HR SEM; Nova Nano SEM 450, Thermo Fisher, Waltham, MA, USA) was used to examine the morphological characteristics of the fabricated AgNPs. For imaging, 1nA was consistently provided for 7 s at 10 kV. Energy dispersive X-ray (EDX) was integrated with HR SEM to examine the elemental makeup of the AgNPs, so that their purity could be predicted. Atomic force microscopy (NT-MDT, Moscow, Russia) was used to study the topological characteristics of green biosynthesized AgNPs in close proximity. To examine surface properties, a drop of AgNP solution was mounted over a small glass slide and dried under a table lamp (200 W). AFM images were analyzed to calculate the size distribution and roughness profile of the AgNPs using the Nova software (version 3.2.5).

There was an error in the original publication. One of the elements was wrongly identified and labeled. A correction has been made to Section 3. Results and Discussion, 3.2. Characterization of AgNPs, 3.2.5. HR-SEM and EDX, Paragraph 1.

A high-resolution scanning electron micrograph depicted the surface morphology of the green-synthesized AgNPs [33]. HR SEM (Nova Nano SEM 450, FEI Company (S.E.A.) PTE, LTD, USA) data analysis revealed that the green-synthesized AgNPs varied in size. The biosynthesized AgNPs were smooth and spherical in shape (Figure 6A). Images showed that the green-synthesized AgNPs formed aggregates (Figure 6B). The aggregation of nanoparticles might be due to proteins, carbohydrates, and glycoproteins present in the plant constituents. The EDX of the green-synthesized AgNPs showed the presence of silver (Ag). The sharp peak at 3 keV indicated the presence of metallic Ag (Figure 6C). Additional peaks (O, Na, and Si) might be because of the glass on which the sample was coated, and C due to the extract.

There was an error in the original publication. Some FTIR-related entries in Table 1, including peak information, have been revised. A correction has been made to Table 1.

## Figure Correction

There was an error in the original publication. There was an error in the structure of quercitrin. A correction has been made to Figure 8.

There was an error in the original publication. The names of the compounds were incorrectly added. A correction has been made to Figure 11.

The authors state that the scientific conclusions are unaffected. This correction was approved by the Academic Editor. The original publication has also been updated.

## Figures and Tables

**Figure 8:**
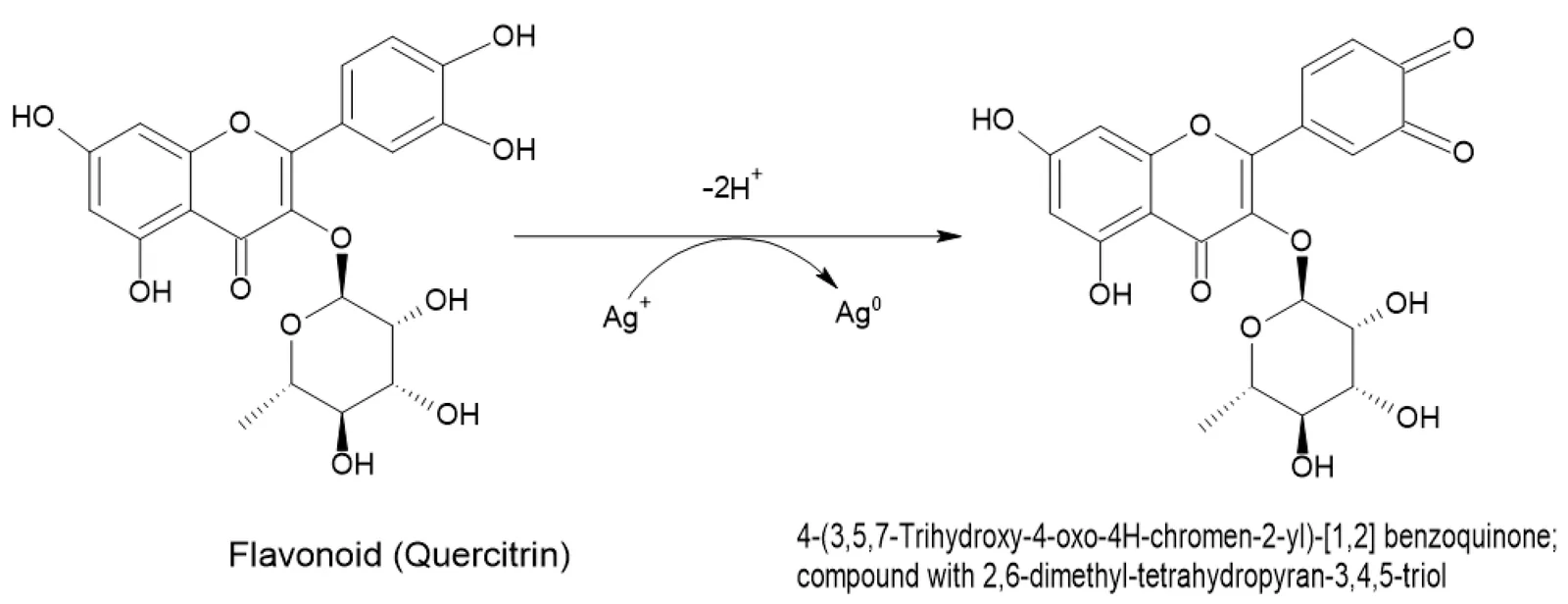
The biosynthesis mechanism of the AgNPs through involvement of the flavonoid (Quercitrin) present in CNAQ.

**Figure 11:**
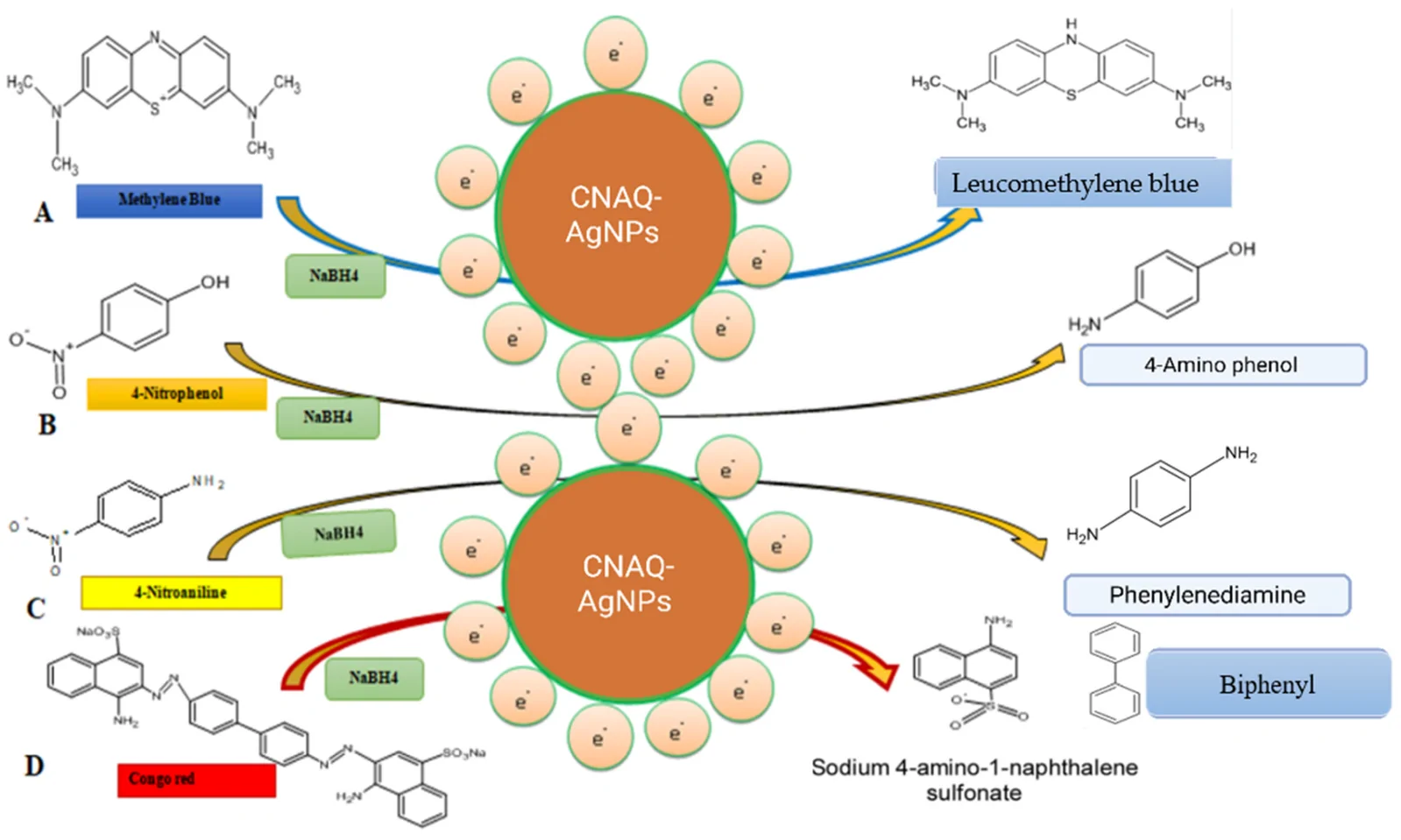
Reduction mechanism of toxic dyes and their catalytic reduction products through the involvement of the AgNPs. (**A**) methylene blue, (**B**) 4-nitrophenol, (**C**) 4-nitroanaline, (**D**) congo red.

**Table 1:** FTIR profiling of CNAQ and AgNPs.

Frequency (cm^−1^)	Peak Wavenumber		Functional Group /Type of Bond	Compounds
	CNAQ	AgNPs		
720–590	596.4	589.5	O-H out-of-plane bend	Hydroxy compounds
1000–600	902 868 819.1	889.5 846.2 794.5	C-H stretching C-H stretching C-H stretching	Alkenes Alkenes Alkenes
1140–1070	1067	1053	C-O stretching	Cyclic ether and oxy group of compounds
1250–1020	1230	1197	C-N- stretching	Aliphatic amines
1420–1300	1302	1287	Carboxylate	Carbonyl compounds
1490–1410	1486	1494	Carbonate ion	Inorganic compounds
1370–1390	1390	1385	N-O stretching	Nitrate ion
1625–1440	1537	1545	C=C stretching	Aromatic compounds
1680–1600	1631	1619	C=C stretching	Alkenes
2000–1650	1761	1777	C-H bending	Aromatic compounds
3200–2500	2990 2920	2984 2920	O-H stretching O-H stretching	Carboxylic acid Carboxylic acid
3550–3200	3416	3410	O-H stretching	Phenol, hydroxy compound
